# An efficient protein extraction method applied to mangrove plant *Kandelia obovata* leaves for proteomic analysis

**DOI:** 10.1186/s13007-021-00800-y

**Published:** 2021-09-29

**Authors:** Jiao Fei, You-Shao Wang, Hao Cheng, Yu-Bin Su

**Affiliations:** 1 State Key Laboratory of Tropical Oceanography, South China Sea Institute of Oceanology, Chinese Academy of Sciences, Guangzhou, 510301 China; 2 Southern Marine Science and Engineering Guangdong Laboratory, Guangzhou, 511458 China; 3 Innovation Academy of South China Sea Ecology and Environmental Engineering, Chinese Academy of Sciences, Guangzhou, 510301 China; 4 College of Life Science and Technology, Jinan University, Guangzhou, 510632 China

**Keywords:** *Kandelia obovata*, Protein extraction, Mangrove, Proteomic, 2-DE

## Abstract

**Background:**

Mangroves plants, an important wetland system in the intertidal shores, play a vital role in estuarine ecosystems. However, there is a lack of a very effective method for extracting protein from mangrove plants for proteomic analysis. Here, we evaluated the efficiency of three different protein extraction methods for proteomic analysis of total proteins obtained from mangrove plant *Kandelia obovata* leaves.

**Results:**

The protein yield of the phenol-based (Phe-B) method (4.47 mg/g) was significantly higher than the yields of the traditional phenol (Phe) method (2.38 mg/g) and trichloroacetic acid-acetone (TCA-A) method (1.15 mg/g). The Phe-B method produced better two-dimensional electrophoresis (2-DE) protein patterns with high reproducibility regarding the number, abundance and coverage of protein spots. The 2-DE gels showed that 847, 650 and 213 unique protein spots were separated from the total *K. obovata* leaf proteins extracted by the Phe-B, Phe and TCA-A methods, respectively. Fourteen pairs of protein spots were randomly selected from 2-DE gels of Phe- and Phe-B- extracted proteins for identification by matrix-assisted laser desorption/ionization time-of-flight mass spectrometry (MALDI-TOF/TOF-MS) technique, and the results of three pairs were consistent. Further, oxygen evolving enhancer protein and elongation factor Tu could be observed in the 2-DE gels of Phe and Phe-B methods, but could only be detected in the results of the Phe-B methods, showing that Phe-B method might be the optimized choice for proteomic analysis.

**Conclusion:**

Our data provides an improved Phe-B method for protein extraction of *K. obovata* and other mangrove plant tissues which is rich in polysaccharides and polyphenols. This study might be expected to be used for proteomic analysis in other recalcitrant plants.

## Introduction

Proteomics has been developed as an important approach for studying plant functional genomics [1], which can be used to detect the post-transcriptional modification [2]. Two-dimensional gel electrophoresis (2-DE) was an efficient and powerful strategy to study complex gene expression at the protein level [3–5]. The quality of protein sample was the most crucial steps for optimal results in proteomic analysis. However, it can be problematic because of co-extraction and other non-protein components [6]. It was even worse in plant tissues due to the relative low content of proteins compared with other components, such as proteases and oxidative enzymes, cell walls and vacuoles, pigments, lipids, starches polysaccharides, organic acids, polyphenols, and other secondary metabolites [7–9]. The protein extraction methods used for processing plant samples may affect subsequent experimental results [10].

Mangroves plants, an important wetland vegetation in the intertidal shores, play a vital role in estuarine ecosystems [11, 12]. On account of high levels of interfering compounds, including tannins [13], which made it difficult to extract proteins from mangrove plants. How to obtain high quality proteins was crucially important for the proteomic study of mangrove plants. Many protocols have been applied to improve plant protein extraction for 2-DE, the method of trichloroacetic acid-acetone (TCA-A) precipitation was a classical strategy for most proteomic studies of in different plant tissues (leaves, roots, fruit, seeds and stems) [8, 14–18]. However, proteins could not be fully redissolved after TCA-A precipitation and some polymeric contaminants were often co-extracted by the TCA-A method [8, 19–21]. Furthermore, when the TCA-A method was used to extract leaf proteins for mangrove plant *Kandelia candel*, obvious vertical stripes and smearing were found in the gels of sodium dodecyl sulfate-polyacrylamide gel electrophoresis (SDS-PAGE) gels [22]. The quality of protein extraction will directly affect the proteomic analysis results. An alternative basic method was phenol (Phe) method. Phenol was observed as an effective agent for extracting proteins from aqueous solutions in some plants [8, 16]. Phe method has been applied to mangrove plants, such as *Avicennia marina* [23], *Bruguiera gymnorhiza* [24], *Rhizophora stylosan* [25], *K. candel* [26, 27] and *K.obovata* [28]. However, the profiles of SDS-PAGE gels in these studies exhibited a high background, indicating the presence of substantial interfering substance. It seemed that the Phe method was not powerful enough to remove interfering compounds for mangrove plants for proteomics research.

There is a lack of a very effective method for extracting protein from mangrove plants for proteomic analysis. *Kandelia obovata*, the most cold-resistant specie of mangrove plants, is widely distributed along the South China Coast [29]. With regard to low-abundance proteins in mangrove plants, we developed an improved protein extraction method (Phe-B) for proteomics studies on mangrove plant *K. obovata* in this study. Comparing with the traditional TCA-A and Phe menthods, the phenol-based (Phe-B) method has been successfully developed for extracting proteins from mangrove plant *K. obovata*. The protein yield and quality were also discussed.

## Results and discussion

### Quantitative comparison of protein yield

**Fig. 1:**
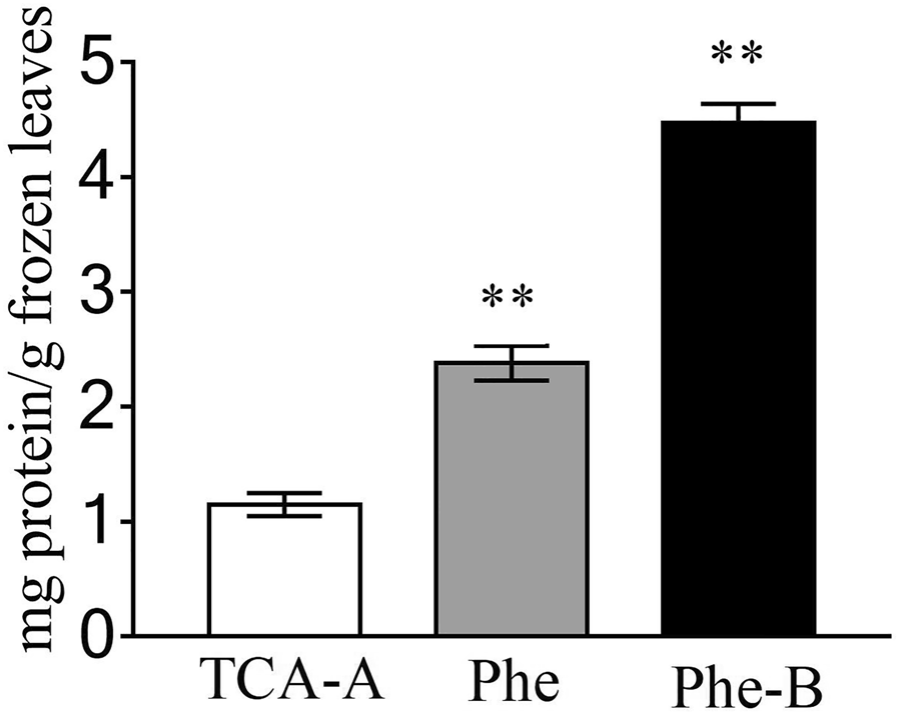
Protein yields of *K. obovata* leaves using three different extraction methods. Values represented the average of three biological replicates. The error bars indicated the standard deviations (*p* values were calculated according to Student’s t test. **p < 0.01)

The protein yields of *K. obovata* leaves obtained by three extraction methods were summarized in Fig. 1. Quantitative comparison of protein extracts revealed that the yield amount of protein by the Phe-B method (4.47 ± 0.17 mg/g) was significantly greater than that by the Phe method (2.38 ± 0.15 mg/g) or TCA-A method (1.15 ± 0.10 mg/g).

### SDS-PAGE evaluation of three methods with *K. obovata* leaves

**Fig. 2:**
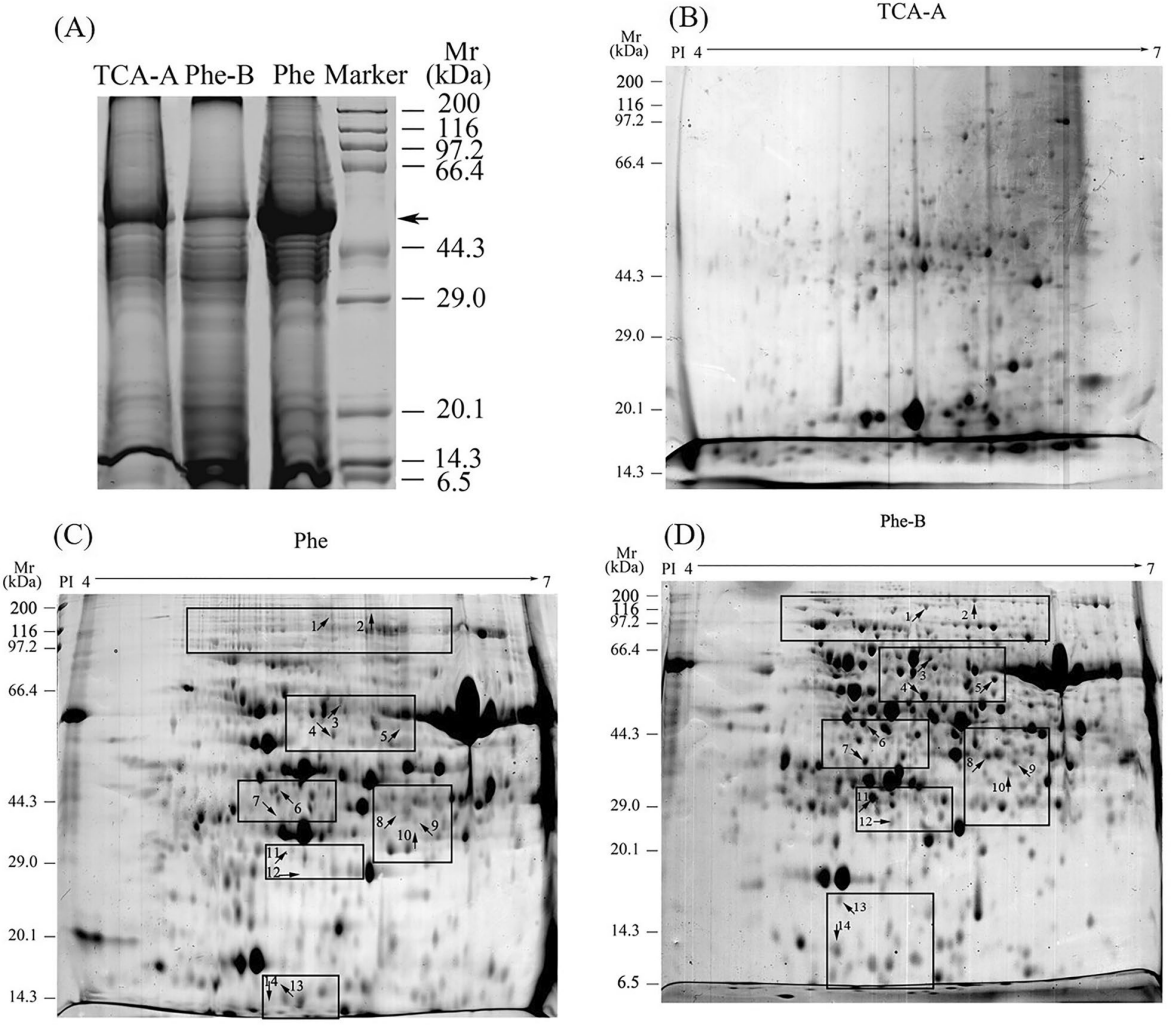
Representative SDS-PAGE and 2-DE gels of the total proteins extracted by three methods. (**A**) SDS-PAGE gel of three protein extracts and protein marker. The black arrow indicated the location of the Rubisco protein. (**B**) 2-DE gel of total proteins extracted by TCA-A method. (**C**) 2-DE gel of total proteins extracted by Phe method. (**D**) 2-DE gel of total proteins extracted by Phe-B method. In all the 2-DE gels, the isoelectric point ranged from 4 to 7. Fourteen pairs of protein spots were randomly selected from the Phe (**C**) and Phe-B (**D**) gels for MALDI TOF/TOF (indicated by arrows and numbers). Due to the failure to obtain a clear 2-DE map, no protein spots were selected from the TCA-A gel (**B**) for MS identification

In Fig. 2A, the three protein extracts from *K. obovata* leaves were performed by SDS-PAGE gel. The *M*_*r*_ of proteins spanned from 6.5 kDa marker to more than 200 kDa. The proteins extracted by Phe-B method had higher quality according to the well-resolved bands distributed in a wide range of *M*_*r*_ (from 6.5 to 116 kD). Besides, proteins extracted by the Phe-B method also showed much less smearing than that by the TCA-A method or Phe method. These results revealed that the Phe-B method was much more effective for protein extraction of *K. obovata* leaves.

There were many differences among the SDS-PAGE bands pattern of the three protein extracts. Particularly, there were much more polypeptides in protein extracts by the Phe-B or Phe methods than that by the TCA-A method. In addition, the obvious difference among the extracts was the relative abundance of Rubisco, as an arrow indicated in Fig. 2A. Protein extracted by Phe-B method contained the lower level of the Rubisco comparing with that by the TCA-A method or Phe method. Rubisco is the world’s most abundant protein in plants and shares more than 50% of the total leaf protein in some species [30]. It can reduce detectable protein spots during electrophoretic separation of leaf proteins. Therefore, several multistep techniques have been developed to remove Rubisco selectively as a pre-fractionation step [31, 32]. The results showed that the Phe-B method was a rapid and efficient method to reduce Rubisco amount. This further indicated that the Phe-B method was the optimal method to acquire high quality proteins from *K. obovata* leaves for proteomics analysis.

### 2-DE evaluation of three methods with *K. obovata* leaves

The proteins extracted by the Phe-B showed better solubility and less streaks than that by the Phe method or TCA-A method. In Fig. 2B–D, protein spots extracted by the Phe-B method (847 ± 93) were more than that by the Phe (650 ± 66) or TCA-A method (213 ± 49). Additionally, spot-to-spot comparison of 2-DE gels revealed that almost all the protein spots extracted by Phe method or TCA-A method were included in that by Phe-B method. These results indicated that the Phe-B method had improved the efficiency of protein extraction and had increased the solubility of proteins. This data further confirmed that the Phe-B method was effective to extract proteins from *K. obovata* leaves.

Compared theses three methods, the Phe-B method obtained the largest numbers of spots, while the TCA-A method got the lowest. Obvious differences were observed in the spot patterns among these extracts. The proteins extracted by Phe-B showed greater spot intensities than that by the Phe method or TCA-A method. The protein spots extracted by the Phe-B and Phe methods were highlighted by black frames in Fig. 2C, D. Based on different protein extraction methods, differential spot patterns reflected different degrees of proteolysis [16]. Table 1 gave the details about the identification of 14 protein spots that are extracted by Phe-B method. Fig. 2C (by Phe method) and Fig. 2D (by Phe-B method) showed the digging locations of these 14 protein spots, respectively. None of these 14 proteins spots were found in Fig. 2B (by TCA-A method). As for mass spectrum analysis, only spot nos. 3, 4, 6 were identified from the protein spots that are extracted both by the Phe (Fig. 2C) and Phe-B method (Fig. 2D). The other 11 spots were identified only in extracts by the Phe-B method (Fig. 2D). However, these missing protein spots, which were lost in the other two extracts, played very important roles in plants. The lost proteins contained aconitase (spot no. 2), adenosine kinase (spot no. 7), fructose-1, 6-bisphosphatase (spot no. 8), thioredoxin reductase (spot no. 10), oxygen evolving enhancer protein (spot no. 11) and ATP synthase (spot no. 13). Previous studies showed that these proteins are involved in the process of metabolism, photosynthesis and anti-stress physiology [33–38].

**Table 1:** Identification of selected protein spots from *K. obovata* leaves by MALDI-TOF/TOF-MS

Spot no.	Genbank accession	Protein identification	Species	Mr (kDa)		p*I*		Score	Expect	Matches	Sequence coverage (%)
				Pred.	Obs.	Pred.	Obs.				
1	gi|672134785	ATP-dependent Clp protease ATP-binding subunit clpA homolog CD4B, chloroplastic	*Phoenix dactylifera*	102	95	6.13	5.65	554	9.00E-50	32	33
2	gi|255566397	aconitase, putative	*Ricinus communis*	109.4	97	7.11	5.9	203	1.10E-14	15	15
3^a^	gi|394831087	ATP synthase CF1 alpha subunit (chloroplast)	*Datura stramonium*	55.49	54	5.15	5.68	545	1.50E-47	18	30
4^a^	gi|297804102	Chloroplast elongation factor tub	*Arabidopsis lyrata subsp. lyrata*	51.87	49	5.84	5.62	267	4.50E-21	8	19
5	gi|659087282	Glutamate--glyoxylate aminotransferase 2	*Cucumis melo*	52.95	51	5.63	6.02	319	2.90E-26	15	28
6^a^	gi|571475731	Putative lactoylglutathione lyase-like isoform X3	*Glycine max*	32.5	31	5.76	5.21	210	2.30E-15	5	13
7	gi|645277823	Adenosine kinase 2	*Prunus mume*	37.96	37	5.22	5.22	248	3.60E-19	7	24
8	gi|568214952	Fructose-1,6-bisphosphatase, cytosolic	*Solanum tuberosum*	37.74	38	5.8	5.98	194	9.00E-14	9	27
9	gi|255566888	Plastid-specific 30S ribosomal protein 1, chloroplast precursor, putative	*Ricinus communis*	35.02	36	9.18	6.15	259	2.90E-20	7	18
10	gi|672158791	Thioredoxin reductase NTRB-like	*Phoenix dactylifera*	35.63	35	5.92	6	83	0.012	4	10
11	gi|8131593	Oxygen evolving enhancer protein 2	*Bruguiera gymnorhiza*	17.58	17	4.91	5.3	181	1.80E-12	5	40
12	gi|657998060	Proteasome subunit beta type-6-like	*Malus domestica*	25.84	24	5.03	5.4	220	2.30E-16	9	33
13	gi|22797141	ATP synthase epsilon subunit	*Olea europaea*	14.68	15	6.51	5.08	128	3.60E-07	4	39
14	gi|587906415	Elongation factor Tu	*Morus notabilis*	52.23	51	6.3	5.05	109	2.90E-05	5	16

Predicted (Pred.) and observed (Obs.) *Mr* and p*I* values and sequence coverage are shown

^a^Peptide spot that is consistent in different extracts

Several studies have involved in the protein extraction of mangrove plant [22–28, 39, 40], including *Rhizophora stylosa*, *Bruguiera parviflora*, *Avicennia marina*, *Bruguiera gymnorhiza*, *Kandelia candel* and *K. obovata*, but these protein extracts showed obvious smearing and streaking in 2-DE gels, and the protein quality were not good enough when applied to proteomics analysis in the studies. In this study, fewer smears and streaks, more spots and higher protein yields were obtained by the Phe-B method (as shown in Figs.1, 2). These results indicated that the Phe-B method was a very good method for the protein extraction of mangrove plants.

As is well-known that most interfering compounds would reduce solubility of proteins and prevent sample powder homogenizing thoroughly in aqueous buffer [41]. Thus, it is very important to remove interfering compounds before protein extraction. Here, the outstanding advantages of the Phe-B method were described as follows: (1) Pulverizing plant tissues with PVPP (0.1 g/g tissue) helps to increase the removal efficiency of phenolic compounds [42, 43]. (2) Briefly washing with 10% TCA/acetone can promote protein precipitation and contaminant removal (most lipids and lipid-like polymers) [44, 45]. Besides, this step also can help to reduce protein degradation and modifications during long-term exposure in low pH (TCA) [21]. (3) Washing with 80% methanol plus 0.1 M ammonium acetate helps to neutralize residual TCA and increase the pH to above 7. The alkaline environment facilitates the subsequent protein extraction by phenol [21]. (4) Phenol/SDS mixture helps to improve protein solubility. As an excellent solubilizing agent, SDS allowed the recovery of membrane-bound proteins [9, 43]. The cautions mentioned in the study were important for obtaining high quality proteins from mangrove plants.

As to comparative proteomics, a major objective is to maximize the number of polypeptides. The Phe-B method obtained the greatest numbers of proteins in comparison with the other two methods. Although the Phe-B method was somewhat complicated and time consuming, a greater number of proteins had been obtained. In addition, the Phe-B method might lose small amounts of proteins due to many steps, but the loss can be remedied by parallel experiments.

## Conclusions

In comparing these three methods, the Phe-B method gave the greatest protein yield, the most protein spots, and the least of smearing and streaking. This is the first time for the well-resolved SDS-PAGE and 2-DE protein patterns of *K. obovata* leaves. The Phe-B method might be the optimal method for extracting proteins from mangrove plants. This study also provides a potential method for protein extraction from other recalcitrant plant tissues for proteomic analysis.

## Methods

### Plant materials and growth

*Kandelia obovata* propagules, collected from Dong-chong mangrove wetland (Shenzhen, China), were used in all the experiments. Propagules were surface sterilized and germinated in clean sands. Seedlings were grown in a greenhouse under a 14 h light/10 h dark cycle at 25/22 ℃, and were irrigated with 1/2 Hoagland solution once a week. After 3 months, two pairs of fully-expanded leaves were collected from seedlings. The leaves were washed with distilled water, and then dried with paper. All the harvested samples were immediately frozen in liquid nitrogen and stored at – 80 ℃ until protein extraction.

### Protein extraction by TCA-A method

The TCA-A method was performed as described [15] with some modifications. Frozen plant leaves (2.0 g) were finely powdered in a pestle and mortar with liquid nitrogen. Three replicate leaf samples were used for protein extraction. The frozen powder was then transferred into a 50 mL centrifuge tube. Filled the tube with four volumes of ice-cold acetone containing 10% TCA and 2% 2-mercaptoethanol. The mixture was adequately homogenized using a vortex mixer and then was precipitated at – 20 ℃ overnight. Then centrifugated the tube at 12,000×*g* for 30 min at 4 ℃ to obtain the protein pellet. The protein pellets were washed with cold acetone, which containing 0.07% 2-mercaptoethanol and 0.1 mM phenylmethanesulfonyl fluoride (PMSF). Then centrifuged the protein pellets at 12,000×*g* for 10 min at 4 ℃ and discarded the supernatant. Repeated this step twice to obtain clean protein pellet. The process of protein extraction by TCA-A method was repeated at least three times.

### Protein extraction by Phe method

Total protein extracts were prepared according to Wang et al. [26]. Frozen plant leaves (2.0 g) were finely powdered in liquid nitrogen, and then suspended completely in 15 mL cold acetone. Three duplicate leaf samples were used for protein extraction. After vibrated the mixture for 1 min, the suspension was centrifuged in a pre-cooling rotor at 12,000×*g* for 5 min at 4 ℃. The supernatant was discarded and the pellet was re-suspended with pre-cooling extraction buffer (100 mM Tris–HCl, pH 8.0, 50 mM L-ascorbic acid, 100 mM KCl, 50 mM disodium tetraborate decahydrate, 1% (v/v) Triton X-100, 2% (v/v) β-mercaptoethanol, and 1 mM PMSF). After adding an equal volume of ice-cold Tris-saturated phenol (pH 7.9), the homogenate was thoroughly mixed and centrifuged at 12,000×*g* for 10 min at 4 ℃. The upper phenolic phase was collected and then was transferred into a new 50 mL tube. The phenol phase was mixed with five volumes of methanol containing 0.1 M ammonium acetate. Then the mixture was precipitated at – 20 ℃ for overnight. After centrifuging the tube at 12,000×*g* for 20 min at 4 ℃, the supernatant was discarded. The protein pellets were washed with cold methanol for one time, and then were washed twice with acetone. During each wash step, mixed and centrifuged them well. The process of protein extraction by TCA-A method was repeated at least three times.

### Protein extraction by the Phe-B method

Based on TCA-A precipitation and phenol extraction with SDS-containing buffer, the Phe-B method was proposed. Three repeated leaf samples were used for protein extraction. Frozen *K. obovata* leaves (2.0 g) were subjected to the follows: (1) tissue powder. After 10% PVPP of sample weight were added, frozen leaves were powdered with liquid nitrogen. (2) TCA/acetone washing. The powder was transferred into a 50 mL tube and then was filled with pre-cooling 10% TCA/acetone. The mixture was extensively homogenized and centrifuged at 12,000×*g* for 5 min at 4 ℃. The supernatant was carefully removed with pipetting. (3) Methanol washing. The pellets were washed by 80% methanol containing 0.1 M ammonium acetate to remove residual TCA (the pH value to above 7). The mixture was mixed well and centrifuged at 12000×*g* for 5 min at 4 ℃. Then discarded the supernatant. (4) Acetone washing. The pellet was washed and vibrated again with 80% acetone until the pellet was fully dispersed. Centrifuged the mixture at 12000×*g* for 5 min at 4 ℃ and discarded the supernatant. (5) Drying. The pellets were freeze-dried for at least 5 min to remove the residual acetone. (6) Protein extraction and precipitation. The protein particles were suspended with 0.4–0.8 mL/0.1 g starting material of 1:1 phenol (pH 7.9)/sodium dodecyl sulfate (SDS) buffer (30% sucrose, 2% SDS, 0.1 M Tris-HCl, pH 8.0, 5% 2-mercaptoethanol, 1 mM PMSF). The mixture was mixed and incubated for 5 min. Then centrifuged the mixture at 12,000×*g* for 10 min and transferred the upper phenol phase into a new 50 mL tube. The upper phenol phase was mixed with five–ten volumes of methanol containing 0.1 M ammonium acetate. The mixture was precipitated at – 20 ℃ for overnight. When a white pellet was visible, centrifuged the mixture at 12,000×*g* for 20 min at 4 ℃ and carefully discarded the supernatant (note: if no phase separation occurs, add more phenol (100 μL) into the mixture, then mixed and centrifuged the mixture again). (7) Washing and air-drying the pellet. The final protein pellet was washed once with 100% methanol and then was washed twice with 80% acetone. During each washing step, the sample should be mixed well and be centrifuged as above. Then discarded the supernatant. The process of protein extraction by Phe-B method was repeated at least three times.

### Protein solubilization and concentration determinations

All the protein pellets by these three protocols were briefly lyophilized by Freeze Dry Systems (SCIENTZ-12N, NINGBO SCIENTZ Biotechnology CO, LTD). The samples were transferred into 1.5 mL microcentrifuge tubes respectively, and were re-dissolved directly in appropriate lysis buffer [7 M urea, 2 M thiourea, 4 % (v/v) CHAPS, 2 % (v/v) pharmalyte 4-7, and 50 mM dithiothreitol (DTT), 2 mM tri-butyl-phosphate (TBP), 0.1 mM PMSF]. Samples were incubated in water bath at 36 ℃ for 2 h, and then was centrifuged at 14,000×*g* for 30 min at 4 ℃. The supernatants were stored at – 80 ℃ for concentration analysis or downstream experiments. The concentrations of protein were quantified by Bradford [46].

### Isoelectric focusing (IEF) and SDS-PAGE

IPG strips (17 cm pH 4-7, Bio-Rad ReadyStrip; Bio-Rad) were passively rehydrated at 17 ℃ for 14 h with 330 μL IEF buffer (7 M urea, 2 M thiourea, 4 % (v/v) 3-[(3-Cholanidopropyl) dimethylammonio]-1-propanesulfonate (CHAPS), 2 % (v/v) pharmalyte 4-7, 50 mM DTT, 2 mM TBP, 0.1 mM PMSF, and 0.002% bromophenol blue) containing 2.0 mg of protein. Isoelectric focusing was performed with a Protean i12 IEF Cell (Bio-Rad) apparatus under the following program: 250 V for 30 min, 500 V for 30 min, 1000 V for 30 min, 8000 V for 5 h and 8000 V for a total of 40,000 Vh. After finished the IEF protocol, the focused strips should be equilibrated in reducing equilibration buffer (6 M urea, 20% w/v glycerol, 2% SDS, and 0.375 mM Tris-HCl, pH 8.8, 2% w/v DTT) as soon as possible. After that the strips were equilibrated in same solution that contained 2.5% w/v iodoacetamide instead of 2.0% DTT. The strips were then transferred to 12% SDS-PAGE gels for second dimension electrophoresis by the Bio-Rad PROTEAN xi 2-D Cell gel system (Bio-Rad, USA). There came to the following program: 50 V for 1 h, and then 200 V for 5.5 h for each strip. The SDS electrophoresis buffer (25 mM Tris-base solution (pH 8.3), 192 mM glycine and 0.1% SDS) was used as working solution.

### Image acquisitions and analysis

The 2-DE gels were stained with Bio-Safe Coomassie Brilliant Blue R-250 (Amresco, USA) and then was scanned by GS-800 Calibrated Densitometer (Bio-Rad, USA). All the 2-DE gel separations were repeated three times. Image analysis was performed using PDQUEST software (Bio-Rad, USA). The automatic default spot analysis was used to edit the spot features with manual correction by combining semiautomatic method. The spots were quantified using the % volume criterion. Three repetitions were executed. Student’s t test was performed for statistical analysis.

### MALDI-TOF/TOF-MS analysis

The selected protein spots were manually excised from stained 2-DE gels for mass spectrometric analysis. All the samples were digested by trypsin and then were analyzed by Ultraflex MALDI-TOF/TOF mass spectrometer (Bruker, Bremen, Germany). The analysis was performed under the control of FlexControl™ 3.3 software (Bruker) with external mass calibration. The mass spectrometer was set to perform data acquisition with a selected mass range of 800–3500 m/z. Internal calibration of the standard spectra was performed after every 10 consecutive spectra using Pepmix peptide calibration standards (Bruker Daltonics). The obtained spectrum was analyzed with FlexAnalysis™ 3.3 (Bruker) and Biotool™ 2.2 (Bruker). Peptide mass fingerprinting was searched using the program Mascot (Matrix Science, London, UK) against the NCBI database. Zero–two peptide cleavage sites were set on the MASCOT search engine. The mass tolerance was 100 ppm, and MS/MS tolerance was 0.6 Da. Protein scores more than 76 were considered statistically significant (p < 0.05) for peptide mass fingerprinting in MS/MS analysis.

## Data Availability

Not applicable.

## Abbreviations

TCA-A: Trichloroacetic acid—acetone method
Phe-B: Phenol-based method
Phe: Phenol method
PMSF: Phenylmethanesulfonyl fluoride
DTT: Dithiothreitol
TBP: Tri-butyl phosphate
MALDI-TOF/TOF-MS: Matrix-assisted laser desorption/ionization tandem time-of-flight mass spectrometry
